# Supplementary material for: Epidemiology of Hereditary Diseases in the Karachay-Cherkess Republic
Source: Int J Mol Sci. 2020 Jan 3;21(1):325. doi: 10.3390/ijms21010325 (PMC6981994; doi:10.3390/ijms21010325)
Supplement: Supplementary file 1 [file ijms-21-00325-s001.pdf]

Supplementary Tables S1–S6

Note: 1 – Ust-Dzhegutinsky district; 2 – Karachaevsky district; 3 – Malokarachaevsky district; 4– Cherkessk City; 5 – Prikubansky district; 6 – Urupsky district; 7 – Zelenchuksky district; 8 – Abazinsky district; 9 – Khabezsky district; 10 – Adyge-Khablsky district; 11 – Nogaysky district; T/I – type of inheritance; AD – autosomal dominant type of inheritance, AR – autosomal recessive type of inheritance, XL – X-linked type of inheritance. PS – Phenotypic Series for OMIM in case of heterogeneity of the disease; Isol. – Isolated cases; Som. – Somatic mutation

Table S1. Nosological spectrum and prevalence (per 100000) of hereditary neurological diseases in Karachay-Cherkess Republic (KChR)

№	OMIM	Diagnosis	T/I	Number of patients												Prevalence (per 100000)						
				1	2	3	4	5	6	7	8	9	10	11	Σ	Karachays	Russian	Circassians	Abazins	Nogais	Others	Σ
1.	#156200	Undifferentiated mental retardation	AD		7	4	4	7				4	6	7	39	12.31	6.68	15.7		13.6		9.50
2.	#249500	Undifferentiated mental retardation	AR	10	10	19	6	23	9	1	2	17	16	20	133	31.40	26.71	41.2	9.02	74.6	76.68	32.41
3.	#309530	Undifferentiated mental retardation	XL	10	8	3	6	18	5	6	3	12	2	3	76	40.63	26.71	19.7	42.1	54.3	125.5	37.04
4.	#300624	Martin-Bell syndrome	XL	2	1	4	3			2	2		1		15	8.62		7.87	36.1			7.31
5.	#158600	Spinal muscular atrophy, juvenile, proximal	AD				2								2		1.48					0.49
6.	#253300	Spinal muscular atrophy, type 1	AR		1	1									2	0.62			3.01			0.49
7.	#253400	Spinal muscular atrophy, type 3	AR			1	2								3	0.62	0.74				6.97	0.73
8.	#159000	Muscular dystrophy, limb-girdle, type 1A	AD		3										3	1.85						0.73
9.	#253600	Muscular dystrophy, limb-girdle, type 2A	AR							2					2		1.48					0.49
10.	#310200	Muscular dystrophy, Duchenne type	XL		1		1			3					5	2.46	1.48	7.87				2.44
11.	#300376	Muscular dystrophy, Becker type	XL			1				1			1		3	1.23	1.48	3.94				1.46
12.	#254090	Ullrich congenital muscular dystrophy	AR							1					1	0.62						0.24
13.	#602771	Muscular dystrophy, rigid spine	AR							1					1	0.62						0.24
14.	#158900	Facioscapulohumeral muscular dystrophy 1	AD		1					1					2	0.62					6.97	0.49
15.	#181430	Scapuloperoneal syndrome, myopathic type	AD							1					1						6.97	0.24
16.	#310400	Myotubular myopathy, X-linked	XL							1					1	1.23						0.49
17.	#161800	Myopathy, actin, congenital, with excess of thin myofilaments	AD							3				1	4		1.48				6.97	0.97
18.	165000	Ophthalmoplegia, familial static	AD		2										2	1.23						0.49
19.	#118220	Charcot-Marie-Tooth disease, type 1A	AD	3	1					4			3	1	12	2.46	2.97	5.90		6.78		2.92
20.	#118210	Charcot-Marie-Tooth disease, type 2A	AD							3					3		2.23					0.73
21.	#607684	Charcot-Marie-Tooth disease, type 2 E	AD	13											13	8.00						3.17
22.	#302800	Charcot-Marie-Tooth neuropathy, X-linked dominant	XL							2			4		6		2.97	15.7				2.92
23.	#256800	Neuropathy, congenital sensory, with anhidrosis	AR	2						1					3	1.85						0.73
24.	#128100	Dystonia, torsion	AD				1			1					2		1.48					0.49
25.	#160900	Myotonic dystrophy 1	AD	2	2								1		5	2.46		1.97				1.22
26.	#159900	Dystonia-myoclonic	AD										1		1			1.97				0.24
27.	#143100	Huntington disease	AD							4		7			11		2.97		21.0			2.68

28.	#164400	Spinocerebellar ataxia 1	AD				1			1					2		1.48					0.49
29.	#141500	Migraine, familial hemiplegic, with progressive cerebellar ataxia	AD											1	1					6.78		0.24
30.	#615369	Epileptic encephalopathy, childhood-onset	AD					3					3		6			5.90				1.46
31.	#612164	Epileptic encephalopathy, early infantile, 4 type	AD		2		5	0	3						10	6.16	0.00					2.44
32.	#607208	Epileptic encephalopathy, early infantile, 6 (Dravet syndrome)	AD							1				1	2		0.74			6.78		0.49
33.	#607876	Epilepsy, familial adult myoclonic	AD							1					1			1.97				0.24
34.	#254800	Myoclonic epilepsy of Unverricht and Lundborg	AR				3			1	1				5	1.85	0.74		3.01			1.22
35.	PS254800	Myoclonic epilepsy	AR							1					1				3.01			0.24
36.	#118800	Paroxysmal nonkinesigenic dyskinesia	AD											1	1					6.78		0.24
37.	#170400	Hypokalemic periodic paralysis, type 1	AD										1		1			1.97				0.24
38.	#191100	Tuberous sclerosis	AD										7		7			13.8				1.71
39.	#162200	Neurofibromatosis, type 1	AD	1	1		1	2	1	1	2	7	1	7	24	3.08	6.68	3.94	6.01	6.78	34.86	5.85
40.	#101000	Neurofibromatosis, type 2	AD		3										3	1.85						0.73
41.	141300	Hemifacial atrophy, progressive	AD		1										1	0.62						0.24
42.	#257320	Lissencephaly 2 (Norman-Roberts type)	AR										2		2			3.94				0.49
43.	#250100	Metachromatic leukodystrophy	AR					1							1		0.74					0.24
44.	#251200	Microcephaly with mental retardation	AR							1	2	5	1	4	13		2.97	7.87	6.01	13.6	6.97	3.17
45.	#608716	Microcephaly, type 5	AR										3		3			5.90				0.73
46.	#617527	Neurodevelopmental disorder with progressive microcephaly, spasticity, and brain anomalies	AR		1										1				3.01			0.24
47.	#182600	Strumpell disease, familial spastic paraplegia	AD							2					2		0.74		3.01			0.49
48.	#270800	Spastic paraplegia autosomal recessive	AR						1						1		0.74					0.24
49.	#236600	Hydrocephalus, nonsyndromic, autosomal recessive	AR					1	2						3		2.23					0.73
50.	#307000	Hydrocephalus, X-Linked	XL		1	1		1							3	2.46	1.48					1.46
51.	#208900	Ataxia-telangiectasia (Louis-Bar syndrome)	AR			1									1	0.62						0.24
52.	#300055	Mental retardation with psychosis, pyramidal signs, and macroorchidism	XL					2							2		2.97					0.97
53.	#309580	Mental retardation-hypotonic facies syndrome, X-linked	XL			1									1	1.23						0.49
54.	#182940	Neural tube defects	AD		1	1				2	1			1	6	0.62	1.48		6.01	6.78		1.46

Table S2. Nosological spectrum and prevalence (per 100000) of hereditary ophthalmic diseases in Karachay-Cherkess Republic

№	OMIM	Diagnosis	T/I	Number of patients												Prevalence (per 100000)						
				1	2	3	4	5	6	7	8	9	10	11	Σ	Karachays	Russian	Circassians	Abazins	Nogais	Others	Σ
1.	#137750	Glaucoma, primary open angle, juvenile-onset	AD	1				3							4	0.62	2.23					0.97
2.	#231300	Glaucoma, congenital; buphthalmos	AR	2							1				3	1.23		1.97				0.73
3.	#116200	Congenital hereditary cataract	AD	12	2	1	1	5	7	5				1	34	6.16	14.10		6.01	13.6	6.97	8.29
4.	PS116200	Congenital zonular cataract	AD							10			3		13	0.62	0.00	19.7			13.94	3.17
5.	PS116200	Congenital hereditary cataract, autosomal recessive	AR		1								3		4	0.62		5.90				0.97
6.	156850	Cataract, congenital, with microphthalmia	AD					2		2					4	1.23	1.48					0.97
7.	#180500	Axenfeld-Rieger syndrome, type 1	AD											1	1					6.78		0.24
8.	#106210	Aniridia	AD							4					4	0.62	2.23					0.97
9.	142500	Heterochromia iridis	AD			2									2	1.23						0.49
10.	129750	Ectopia pupillae	AD										1		1			1.97				0.24
11.	#120200	Coloboma, ocular	AD	1				1		1					3	0.62	1.48					0.73
12.	#216820	Coloboma, ocular, autosomal recessive	AR					1							1		0.74					0.24
13.	#614497	Microphthalmia, isolated, with coloboma	AD							4					4							0.97
14.	251505	Microphthalmia with coloboma	AR	1			1	1							3	1.23	0.74					0.73
15.	#611040	Microphthalmia, isolated, type 5	AR			1	1	1						1	4	1.23	0.74					0.97
16.	PS251600	Microphthalmia, isolated, type 1	AR		1		3		1				1		6	3.08	0.00	1.97				1.46
17.	#309300	Megalocornea 1, X-linked	XL		1										1	1.23						0.49
18.	164100	Nystagmus, congenital, autosomal dominant	AD			3									3	1.85						0.73
19.	#310700	Nystagmus, congenital, X-linked	XL										1		1					13.6		0.49
20.	#110100	Blepharophimosis, epicanthus inversus, and ptosis	AD							1					1		0.74					0.24
21.	#178300	Ptosis, hereditary congenital	AD	3		1	1	2			3	1	4		15	4.31	0.74	7.87	9.02			3.66
22.	#148300	Keratoconus	AD					1	1		2		6		10	0.62	0.74	11.8	6.01			2.44
23.	#180100	Retinitis pigmentosa	AD		4							1			5	3.08	0.00					1.22
24.	#613341	Retinitis pigmentosa, juvenile	AR										1		1			1.97				0.24
25.	#312600	Retinitis pigmentosa, X-linked	XL	1											1	1.23						0.49
26.	#153700	Macular dystrophy, Best disease	AD					1	3			2			6	0.00			6.01			1.46
27.	#248200	Macular degeneration, juvenile (Stargardt disease)	AR	1	1		2	4	2	2	3	2			17	4.31	2.97	5.90	6.01		6.97	4.14

28.	#303100	Tapetochoroidal dystrophy, progressive	XL							1		1			2	2.46						0.97
29.	#165300	Optic atrophy with cataract	AD				1								1	0.62						0.24
30.	#165500	Optic atrophy	AD							5					5			9.84				1.22
31.	#165550	Optic nerve hypoplasia	AD			5		3		1			1		10	5.54		1.97				2.44
32.	#206900	Optic nerve hypoplasia and abnormalities of the central nervous system	AR							3					3		0.00	5.90				0.73
33.	258500	Optic atrophy, congenital or early infantile, autosomal recessive	AR	1											1	0.62						0.24
34.	#204000	Leber congenital amaurosis	AR	4		1									5	3.08						1.22
35.	#120970	Cone-rod retinal dystrophy	AD				4		2						6	2.46	1.48					1.46
36.	#182230	Septooptic dysplasia	AD		1									1	2	0.62				6.78		0.49
37.	#217800	Macular corneal dystrophy	AR					1							1	0.62						0.24
38.	#257270	Night blindness, congenital stationary, autosomal recessive	AR											1	1					6.78		0.24
39.	#601777	Retinal cone dystrophy	AR		2	1									3	1.85						0.73
40.	#180200	Retinoblastoma	AD	1		1			1	4			1		8	1.23	0.74	1.97	12.0			1.95
41.	#264800	Pseudoxanthoma elasticum	AR				1	1							2	0.62	0.74					0.49
42.	#303800	Colorblindness, partial, deutan series	XL				2								2	2.46						0.97

Table S3. Nosological spectrum and prevalence (per 100000) of hereditary genodermatoses diseases in Karachay-Cherkess Republic

№	OMIM	Diagnosis	T/I	Number of patients												Prevalence (per 100000)						
				1	2	3	4	5	6	7	8	9	10	11	Σ	Karachays	Russian	Circassians	Abazins	Nogais	Others	Σ
1.	#146700	Ichthyosis vulgaris	AD	9	1	5	1		1	10				3	30	4.92	8.16	5.90	9.02	20.4	13.94	7.31
2.	#308100	Ichthyosis, X-linked	XL	1			3			2	2		3	3	14	4.92	2.97	15.7	6.01	40.7		6.82
3.	#242100	Ichthyosiform erythroderma, Brocq congenital, nonbullous form	AR	1	2		1	5		1					10	4.92	1.48					2.44
4.	#148700	Keratosis palmoplantaris striata I	AD	4	25	12		10		10			16	2	79	24.01	2.97	31.5	42.1	13.6	27.88	19.25
5.	151900	Lipomatosis, familial multiple	AD	2	14										16	9.85						3.90
6.	#131900	Epidermolysis bullosa simplex, Koebner type	AD						1						1		0.74					0.24
7.	#129400	Ectodermal dysplasia, anhidrotic, with cleft lip/palate (Rapp-Hodgkin syndrome)	AD	1											1	0.62						0.24
8.	#129490	Ectodermal dysplasia 10A, hypohidrotic/hair/nail type	AD	1	2								2		5	1.85		3.94				1.22
9.	#124200	Darier-White disease	AD											3	3					20.4		0.73
10.	#172800	Piebaldism	AD										1		1			1.97				0.24
11.	104110	Alopecia, familial focal	AD								2				2			3.94				0.49
12.	#162900	Nevus, epidermal, somatic	AD/ Isol.										4		4			7.87				0.97
13.	#305600	Focal dermal hypoplasia	XL					2							2		2.97					0.97
14.	#305100	Ectodermal dysplasia 1, hypohidrotic, X-linked	XL			1									1						13.94	0.49

Table S4. Nosological spectrum and prevalence (per 100000) of hereditary skeletal diseases in Karachay-Cherkess Republic

№	OMIM	Diagnosis	T/I	Number of patients												Prevalence (per 100000)						
				1	2	3	4	5	6	7	8	9	10	11	Σ	Karachays	Russian	Circassians	Abazins	Nogais	Others	Σ
1.	#100800	Achondroplasia	AD	2						2				1	5	0.62	1.48		3.01	6.78		1.22
2.	#146000	Hypochondroplasia	AD		2			2		1		1		1	7	2.46	0.74			6.78	6.97	1.71
3.	#156530	Metatropic dysplasia	AD								1				1			1.97				0.24
4.	156232	Mesomelic dysplasia	AD							1					1		0.74					0.24
5.	#183900	Spondyloepiphyseal dysplasia, congenital type	AD							2					2	0.00	1.48					0.49
6.	#184250	Spondyloepimetaphyseal dysplasia	AD									1			1	0.62						0.24
7.	#271640	Spondyloepimetaphyseal dysplasia with joint laxity	AR	2											2	1.23						0.49
8.	#133700	Multiple cartilaginous exostoses	AD					1		1	3			1	6	0.62	0.74		9.02	6.78		1.46
9.	#183600	Split hand/foot malformation	AD		1			3		1			4		9	1.23	2.23	7.87				2.19
10.	609815	Zygodactyly, type 1	AD							5					5							1.22
11.	#186200	Syndactyly, type IV	AD	1						1					2		0.74		3.01			0.49
12.	#186000	Syndactyly, type II	AD			1									1	0.62						0.24
13.	#185900	Syndactyly, type 1 (Zygodactyly)	AD/ Isol.		2	2				5					9	2.46	0.00					0.97
14.	#108120	Arthrogryposis multiplex, distal, type 1	AD		1				1						2	0.62	0.74					0.49
15.	#174200	Polydactyly, postaxial	AD	1	2		1			3	2			1	10	2.46	0.74	1.97	12.0			2.44
16.	#174500	Polydactyly, preaxial type II	AD					2			1	1			4		1.48		6.01			0.97
17.	#113000	Brachydactyly, type B1	AD										1		1			1.97				0.24
18.	#113200	Brachydactyly, type D	AD					2							2	1.23						0.49
19.	#610713	Brachydactyly-syndactyly syndrome	AD											1	1					6.78		0.24
20.	181800	Scoliosis, idiopathic 1	AD/ Isol.					1	1	2	2	1	2		9		1.48	7.87	6.01		6.97	2.19
21.	#166200	Osteogenesis imperfecta	AD					8	1				2		11	0.62	5.94	3.94				2.68
22.	#104530	Amelogenesis imperfecta, type IA	AD											4	4					27.1		0.97
23.	184400	Sprengel deformity	AD/ Isol.					1							1	0.62						0.24
24.	#136760	Frontonasal dysplasia 1	AR					3							3	0.62	1.48					0.73

25.	#193700	Arthrogryposis, distal, type 2A (Freeman-Sheldon syndrome)	AD	1						1					2	0.62					6.97	0.49
26.	609655	Talo-patello-scaphoid osteolysis, synovitis, and short fourth metacarpals	AR										1		1			1.97				0.24
27.	#249700	Langer mesomelic dysplasia	AR										2		2			3.94				0.49
28.	#277300	Spondylocostal dysostosis, autosomal recessive	AR	2											2	1.23						0.49
29.	217100	Constricting bands, congenital	AR/ Isol.	3	2	1		1					2		9	2.46	0.74	3.94	6.01			2.19

Table S5. Nosological spectrum and prevalence (per 100000) of hereditary syndromes in Karachay-Cherkess Republic

№	OMIM	Diagnosis	T/I	Number of patients												Prevalence (per 100000)						
				1	2	3	4	5	6	7	8	9	10	11	Σ	Karachays	Russian	Circassians	Abazins	Nogais	Others	Σ
1.	#148210	Keratitis-ichthyosis-deafness syndrome	AD			1									1	0.62						0.24
2.	#148350	Keratoderma, palmoplantar, with deafness	AD											1	1					6.78		0.24
3.	#120433	Coloboma, ocular, with or without hearing impairment, cleft lip/palate, and/or mental retardation	AD	1											1	0.62						0.24
4.	192350	VATER association (vertebral defects, anal atresia, tracheoesophageal fistula with esophageal atresia, and radial or renal dysplasia)	AD/ Isol.				1								1	0.62						0.24
5.	#113620	Branchiooculofacial syndrome	AD							3					3		2.23					0.73
6.	156620	Syndrome of microcephaly, deafness/malformed ears, mental retardation	AD							1					1		0.74					0.24
7.	#151100	LEOPARD syndrome (multiple lentigines, electrocardiographic conduction abnormalities, ocular hypertelorism, pulmonic stenosis, abnormal genitalia, retardation of growth, and sensorineural deafness)	AD								1	1			2				3.01		6.97	0.49
8.	#101200	Apert syndrome	AD	1											1				3.01			0.24
9.	#193500	Waardenburg syndrome, type 1	AD			3									3	1.85						0.73
10.	164210	Goldenhar syndrome	AD				2	1		1			1		5	1.85	0.74	1.97				1.22
11.	#147920	Kabuki syndrome	AD	1						1					2	1.23						0.49
12.	149000	Klippel-Trenaunay-Weber syndrome	AD/ Isol.		1							1		1	3	0.62			3.01	6.78		0.73
13.	#118100	Klippel-Feil syndrome	AD		1							1			2	0.62	0.74					0.49
14.	173800	Poland syndrome	AD		1			1							2	0.62	0.74					0.49
15.	#122470	Cornelia de Lange syndrome	AD								1	1			2	0.62			3.01			0.49
16.	#154700	Marfan syndrome	AD		2								2		4	1.23		3.94				0.97
17.	157900	Moebius syndrome	AD/ Isol.		2					1					3	1.23	0.74					0.73

18.	156000	Meniere disease	AD				1								1	0.62						0.24
19.	#163950	Noonan syndrome	AD		1	1	1			1	1				5	1.85	0.74		3.01			1.22
20.	#176920	Proteus syndrome, somatic	Som.	1											1	0.62						0.24
21.	#101600	Pfeiffer syndrome	AD											1	1					6.78		0.24
22.	#187300	Telangiectasia, hereditary hemorrhagic (Osler-Rendu-Weber disease)	AD		1										1	0.62						0.24
23.	#180849	Rubinstein-Taybi syndrome	AD		1	1									2	1.23						0.49
24.	#101400	Saethre-Chotzen syndrome	AD	1											1		0.74					0.24
25.	#117550	Sotos syndrome	Isol.							1					1	0.62						0.24
26.	#108300	Stickler syndrome	AD		2										2	1.23						0.49
27.	#185300	Sturge-Weber syndrome	Som/Isol.			1	2		1			1			5	2.46					6.97	1.22
28.	#142900	Holt-Oram syndrome	AD	1			1	1							3	1.85						0.73
29.	#130000	Ehlers-Danlos syndrome	AD	25	31	36	35	9	6	22	20	8	36	46	274	83.11	20.78	74.8	72.2	257	76.7	66.77
30.	#218600	Craniosynostosis with radial defects (Baller-Gerold syndrome)	AR			1									1	0.62						0.24
31.	#143500	Hyperbilirubinemia, Gilbert syndrome	AR		3	2		1		1			5	2	14	3.69	0.74	9.84		13.57		3.41
32.	#213300	Joubert-Boltshauser syndrome (cerebellooculorenal syndrome)	AR		1	2									3	1.85						0.73
33.	#216550	Cohen syndrome	AR			2									2	1.23						0.49
34.	#245600	Larsen syndrome	AR		1										1	0.62						0.24
35.	#244400	Kartagener syndrome	AR		1								1		2	0.62		1.97				0.49
36.	261800	Pierre Robin sequence	AR/Isol.		3		1						1	1	6	2.46		1.97		6.78		1.46
37.	#274600	Pendred syndrome	AR		1					2		1			4	1.23	0.74				6.97	0.97
38.	#210600	Seckel syndrome	AR		1	2									3	1.85						0.73
39.	#276900	Usher syndrome	AR					2					1		3			1.97				0.73
40.	234100	Hallermann-Streiff syndrome	AR							1					1		0.74					0.24
41.	#600155	Hirschsprung disease-mental retardation syndrome (Mowat-Wilson syndrome)	AR			2									2	1.23						0.49
42.	251800	Microtia with meatal atresia and conductive deafness	AR					2		4				1	7	1.23	2.23	1.97		6.78		1.71
43.	#222300	Optic atrophy and deafness with mental retardarion (Wolfram syndrome)	AR	2										1	3	1.23				6.78		0.73

44.	252100	Orofaciodigital syndrome II (Moro syndrome)	AR/ Isol.						2	2					4	1.23		3.94				0.97
45.	260150	Palant cleft palate syndrome	AR			1									1	0.62						0.24
46.	#210720	Microcephalic osteodysplastic primordial dwarfism, type II	AR			1									1						6.97	0.24
47.	212540	Cataract, microcephaly, arthrogryposis, kyphosis syndrome (Camfak syndrome)	AR										1		1					6.78		0.24
48.	#214150	Cerebrooculofacioskeletal syndrome (COFS syndrome)	AR	1	1										2	0.62						0.24
49.	#220600	Deafness, congenital, with split hands and feet	AR										1		1			1.97				0.24
50.	#236670	Cerebroocular dysplasia-muscular dystrophy syndrome	AR	1											1				3.01			0.49
51.	#261540	Peters-plus syndrome (Peters anomaly with short-limb dwarfism)	AR		1										1	0.62						0.24
52.	#228930	Fuhrmann syndrome (fibular aplasia or hypoplasia, femoral bowing and poly-, syn-, and oligodactyly)	AR			1			1						2	0.62	0.74					0.49
53.	#305400	Aarskog-Scott syndrome	XL								6		2		8			7.87	36.1			3.90
54.	#301050	Alport syndrome	XL							1					1		1.48					0.49
55.	#303600	Coffin-Lowry syndrome	XL	1											1	1.23						0.49
56.	#312750	Rett syndrome	XL	1						1			1	1	4		2.97	3.94		13.6		1.95
57.	#300707	Toe syndactyly, telecanthus, and anogenital and renal malformations (STAR syndrome)	XL	1											1	1.23						0.49
58.	301815	Arthrogryposis, ectodermal dysplasia, cleft lip/palate, and developmental delay	XL								1				1				6.01			0.49
59.	#176270	Prader-Willi syndrome	Isol.	1						5					6		3.71					1.22
60.	#105830	Angelman syndrome	Isol.	1	3					1		1			6	1.85	0.74				6.97	1.22
61.	#180860	Silver-Russell syndrome	Isol.	1		2				1	1				5		0.74		3.01			0.49
62.	#194050	Williams-Beuren syndrome	Isol.							1	1				2		0.74		3.01			0.49

Table S6. Nosological spectrum and prevalence (per 100000) of other hereditary diseases in Karachay-Cherkess Republic

№	OMIM	Diagnosis	T/I	Number of patients												Prevalence (per 100000)						
				1	2	3	4	5	6	7	8	9	10	11	Σ	Karachays	Russian	Circassians	Abazins	Nogais	Others	Σ
1.	#601544	Deafness, autosomal dominant	AD				4						1	1	6	2.46	0.74	1.97				1.46
2.	#220290	Deafness, autosomal recessive 1A	AR	24	30	9	10	13	5	86	13	14	12	7	223	43.09	55.66	41.3	72.2	47.5	181.3	54.34
3.	#304400	Deafness, autosomal X-linked 2	XL											2	2	0.00				13.6		0.97
4.	166800	Otosclerosis	AD	1		1	1								3	1.85						0.73
5.	#606952	Albinism, oculocutaneous, type IB	AD						4						4		2.97					0.97
6.	#203100	Albinism, oculocutaneous, type IA	AR		4	1		3	1	2			1		12	5.54		3.94			6.97	2.92
7.	#106100	Angioedema, hereditary	AD			4									4	2.46						0.97
8.	#173900	Polycystic kidney disease	AD	4											4	2.46						0.97
9.	#173100	Growth hormone deficiency, isolated	AD		8										8	4.92						1.95
10.	#262400	Growth hormone deficiency, isolated, type IA	AR		2		1	1		2	2		1		9	2.46	1.48	3.94	3.01			2.19
11.	#249100	Familial mediterranean fever	AR			1									1	0.62						0.24
12.	#274400	Thyroid dyshormonogenesis	AR	5	9	8	4	3		1	2		2		34	15.39	2.23	5.90	9.02			8.29
13.	#202010	Adrenal hyperplasia, congenital, due to 11-beta-hydroxylase deficiency	AR		1					1	2			1	5	1.23			6.01	6.78		1.22
14.	#230400	Galactosemia	AR	1		1	1						1		4	2.46						0.97
15.	#176000	Porphyria	AD		1										1	0.62						0.24
16.	#261600	Phenylketonuria /Hyperphenylalaninemia	AR	26	14	9	10	9		7	1		4		80	41.86	4.45	3.94	12.0			19.49
17.	#219700	Cystic fibrosis	AR	5	4	7	1			4	1	1			23	11.70	2.23		3.01			5.60
18.	#607014	Mucopolysaccharidosis, type Ih (Hurler syndrome)	AR	1											1		0.74					0.24
19.	#257220	Niemann-Pick disease, type C1	AR							2					2	0.62	0.74					0.49
20.	#230800	Gaucher disease	AR							1					1	0.62						0.24
21.	#304800	Diabetes insipidus, nephrogenic	XL										3		3			11.8				1.46
22.	#613695	Long QT syndrome 5	AD						2						2		1.48					0.49
23.	#307800	Hypophosphatemic rickets, X-linked dominant	XL				1								1	1.23						0.49
24.	132500	Epistaxis, hereditary	AD										2		2			3.94				0.49
25.	#193400	von Willebrand disease, type 1	AD	2	9	1		1		3					16	9.23	0.74					3.90
26.	#182900	Spherocytosis, hereditary type 1	AD										3		3			5.90				0.73
27.	#300908	Hemolytic anemia, G6PD deficient (favism)	XL											5	5					67.8		2.44
28.	#306900	Hemophilia B	XL					3		2					5	1.23	4.45		6.01			2.44

29.	#306700	Hemophilia A	XL	2		5			2	7			1		17	11.08	8.90	3.94	6.01			8.29
